# The association between transforming growth factor beta1 polymorphism and susceptibility to pulmonary fibrosis: A meta-analysis (MOOSE compliant)

**DOI:** 10.1097/MD.0000000000011876

**Published:** 2018-09-14

**Authors:** Lili Xin, Miao Jiang, Guangbao Su, Miao Xie, Hui Chen, Xiao Liu, Muge Xu, Geng Zhang, Jiening Gong

**Affiliations:** Basic Medical College, Nanjing University of Traditional Chinese Medicine, Nanjing City, Jiangsu Province, China.

**Keywords:** genetic polymorphism, pulmonary fibrosis, rs1800470, transforming growth factor beta1 +869C

## Abstract

Although many studies have investigated the association of single nucleotide polymorphisms (SNPs) in transforming growth factor beta1 (TGF-β1) gene with pulmonary fibrosis (PF), but their association is still controversial. To clarify this, we performed a meta-analysis.

Studies related to TGF-β1 and PF were retrieved from PubMed, Medline, Embase, Scopus, and Wanfang (up to November 30, 2017). We targeted TGF-β1 SNPs that have been reported by ≥3 studies to be included in the current meta-analysis, resulting in only 1 final SNP (rs1800470). The odds ratios (ORs) and 95% confidence intervals (CIs) were estimated in the models of allele comparison (T vs C), homozygote comparison (TT vs CC), dominant (TT vs TC + CC), recessive (TT + TC vs CC) to evaluate the strength of the associations.

A total of 7 case-control studies were included in this meta-analysis. Overall, no significant association between TGF-β1 rs1800470 and PF was found (T vs C: OR [95% CI] = 0.96 [0.80, 1.15]; TT vs CC: 0.87 [0.61, 1.22]; TT vs TC + CC: 0.80 [0.62, 1.04]; TT + TC vs CC: 1.13 [0.83, 1.54]). In subgroup analyses by ethnicity or original disease, no statistically significant association between TGF-β1 rs1800470 polymorphisms and PF was demonstrated.

This meta-analysis revealed that TGF-β1 rs1800470 polymorphism was not associated with susceptibility to PF development.

## 1 Introduction

Pulmonary/lung fibrosis or fibrosing alveolitis is an irreversible accumulation of connective tissue in the interstitial and airway of the lung. The pathogenesis of pulmonary fibrosis is complex and remains poorly understood. Additionally, lung fibrosis can result from many pathological processes, including idiopathic interstitial pneumonia (IPF) which is the best-known entity,^[1,2]^ infection, malignancy, and surgical procedures.^[3–5]^ It is widely believed that genetic factors play an important role in the aetiopathogensis of many pulmonary fibrotic disorders.^[6]^ There is also evidence that the development of most fibrosing lung diseases occurs in susceptible individuals and multiple genetic loci, each exerting variable relatively small effects, are involved.^[7–9]^

Transforming growth factor-β1 is a multifunctional cytokine that plays a role in several biological processes and is associated with susceptibility to various diseases, contributing to the influx and activation of inflammatory cells, the epithelial to mesenchymal trans-differentiation of cells and the influx of fibroblasts and their subsequent elaboration of extracellular matrix.^[10,11]^ Furthermore, transforming growth factor beta1 (TGF-β1) polymorphism presented a higher TGF-β expression and was also shown to be associated with increased invasive breast cancer.^[12,13]^ To date, 8 single-nucleotide polymorphisms (SNPs) have been shown to affect TGF-β1 expression (rs2317130, rs11466313, rs1800468, rs1800469, rs11466314, rs1800471, rs1800470, and rs11466316).^[14]^

Similarly, TGF-β1 plays a central role in pulmonary fibrosis. It is the chemotactic for fibroblasts, induces the synthesis of matrix proteins and glycoproteins, and inhibits collagen degradation by induction of protease inhibitors and reduction of metalloproteases.^[15,16]^ In light of the relevance of TGF-β1 to the development of fibrosis, there are a large number of studies conducted to investigate the association of TGF-β1 polymorphisms with the development of pulmonary fibrosis. While some studies have shown that there was a positive association between TT genotype of TGF-β1 rs1800470 and pulmonary fibrosis development,^[17]^ another study found that the CC genotype was a risk factor for susceptibility to IPF.^[18]^

In this study, we performed a meta-analysis to evaluate the association of TGF-β1 rs1800470 polymorphism with pulmonary fibrosis.

## 2 Materials and methods

### 2.1 Search strategy

We searched PubMed, Medline, Embase, Scupos, and Wanfang (up to November 30, 2017) for studies that have evaluated the association of TGF-β1 polymorphism with pulmonary fibrosis in humans using the following search terms:(“pulmonary fibrosis” or “lung fibrosis” or “Alveolitis, Fibrosing” or “fibrosing Alveolitides”) and (“genetic polymorphism” or “genetic variation” or “genetic variant” or “polymorphism” or “variant”) and (“transforming growth factor beta1” or “TGF-beta1”). References cited in relevant articles produced from the search were also manually searched for possible misses in our inclusion. To avoid any potential publication bias, we didn’t place any limitations on publication date, publication type, sample origin, country, or language.

This study has been approved by the ethics committee of Nanjing University of Traditional Chinese Medicine and written informed consent was obtained from all subjects.

### 2.2 Inclusion and exclusion criteria

The inclusion criteria of literature were as follows: case-control design, all patients were diagnosed with pulmonary fibrosis, according to a high-resolution computer tomography (HRCT) of the lung or biopsy and the diagnosis of idiopathic pulmonary fibrosis was based on the official the American Thoracic Society (ATS) and/or the European Respiratory Society (ERS) statement. Providing adequate summary statistics on TGF-β1 genetic variants, and investigating TGF-β1 polymorphism and pulmonary fibrosis, including idiopathic pulmonary fibrosis and other pulmonary fibrotic diseases which could be caused by severe acute respiratory system (SARS), emphysema, cystic fibrosis, and cryptogenic fibrosing alveolitis.

The exclusion criteria were:

(a) studies reporting overlapping data;
(b) studies with incomplete data;
(c) studies with duplicated data.

### 2.3 Data extraction

The data were checked and extracted by 2 investigators independently. The following information were extracted from eligible studies whenever available: first author, publication year, sample size for control and case group, mean age, sex ratio, ethnicity, geographic location, diagnostic tools for pulmonary fibrosis and idiopathic pulmonary fibrosis, and genotype frequency. Disagreement were resolved by discussion among all authors until consensus was reached.

### 2.4 Statistical analysis

The meta-analysis was performed using the Review Manager (RevMan) 5.3 software (Copenhagen: The Nordic Cochrane Centre, The Cochrane Collaboration, 2014) and STATA program (Stata Software Package version 14, College Station, TX). The relationship between TGF-β1 rs1800470 polymorphism and PF was assessed by determining the pooled ORs and 95% CIs for allele (T vs C) and homozygote comparisons (TT vs CC) and for dominant (TT vs TC + CC) and recessive (CC vs TC + TT) models. A random-effect was used to estimate the pooled ORs and 95% CIs, as heterogeneity was found with *I*^2^ > 10%. Statistical significance is set at below 0.05 threshold level. Between-study heterogeneity was evaluated using *I*^2^ statistics. The range of *I*^2^ values quantifies the between-study variability present not owing to random chance; they have the following distribution: 0% to 25% = low heterogeneity, 25% to 50% = moderate heterogeneity, 50% to 75% = large heterogeneity, and 75 to 100 = considerable heterogeneity. When *I*^2^ surpasses 50% threshold, it is warranted to use additional meta-analysis techniques such as subgroup analysis or meta-regression to explain the variance between studies. Publication bias was examined by means of Eggger linear regression test and rank correction test.

## 3 Results

### 3.1 Literature search

**Figure 1:**
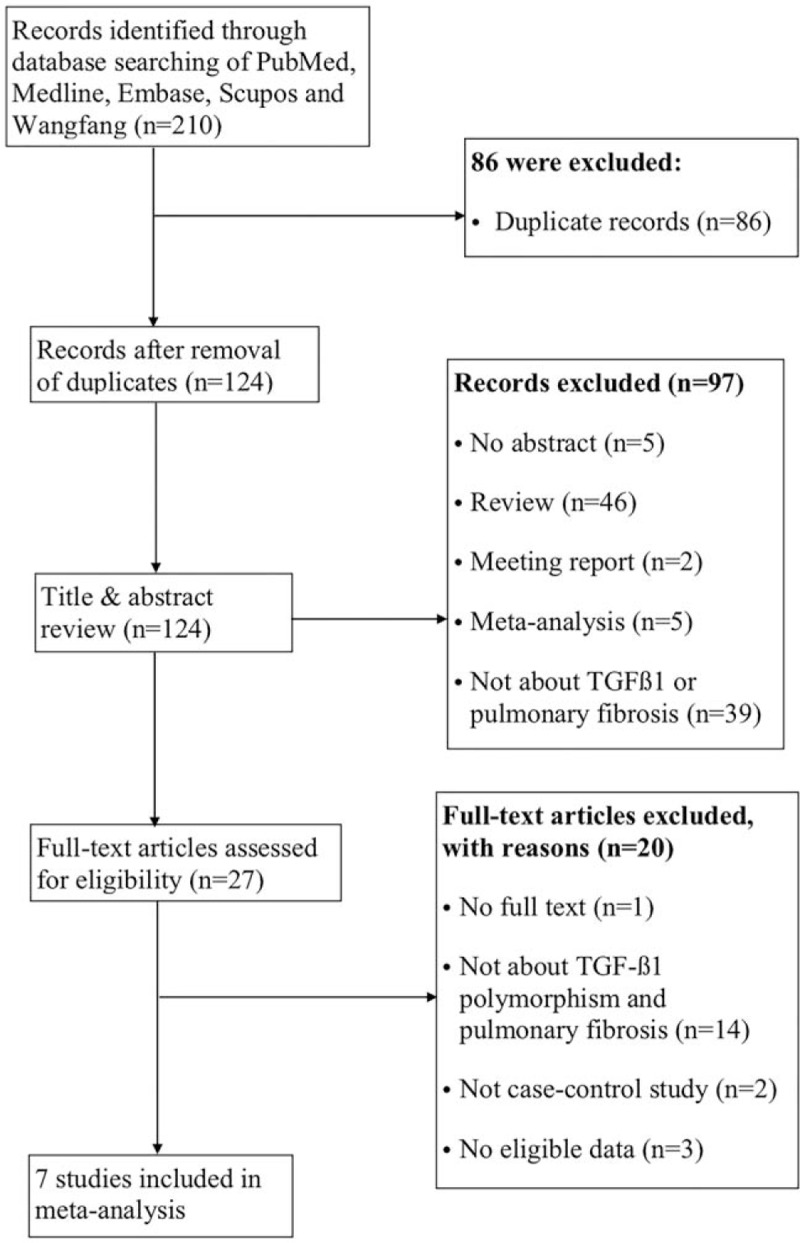
PRISMA flow diagram for inclusion and exclusion of studies in the meta-analysis. PRISMA = preferred reporting items for systematic reviews and meta-analyses.

Figure 1 displays our strategy for literature search. After a comprehensive literature search, a total of 210 studies were initially retrieved including 86 duplicate records. After reviewing the abstracts of 124 non-duplicate studies, we removed 97 studies due to the following reason: the study was not about TGF-β1 or pulmonary fibrosis (n = 39); they did not have an abstract (n = 5); or they were not the original research articles (n = 53), that is, if they were reviews (n = 46), meeting reports (n = 2), or meta-analysis (n = 5). Of the remaining 27 studies, one study was excluded as the full-text article was not available. We further removed 19 studies because of the following reasons: they did not provide detailed information on cases and controls (n = 2); they did not study the association between TGF-β1 and pulmonary fibrosis (n = 14); or they did not provide reusable data (n = 3). Any TGF-β1 SNPs that were reported by 2 or fewer findings were omitted from our selection owing to insufficient data. Our process resulted in 7 applicable studies covering one TGF-ß1 SNP (rs1800470) for the current analysis. The following SNP was excluded owing to limited publications or insufficient data (rs1800471).

### 3.2 Study selection and characteristics

Figure 1 shows the literature search and study selection process. A total of 209 potential publications were identified by our search terms performed on 5 online-databases. During our subsequent literature screening process, we retained only publications relevant to human subjects in case-control studies with sufficient reporting data on TGF-β1 variants. Any non-primary research papers such as review or meta-analysis were manually surveyed and removed. Any TGF-ß1 SNP that was reported by 2 or fewer findings was omitted from our selection owing to insufficient data. In the remaining filtering steps, our preceding inclusion and exclusion criteria were strictly applied. Our process resulted in 7 applicable studies covering only one eligible TGF-β1 SNP (rs1800470).

### 3.3 Characteristics of the studies

All suitable publications were published between 1998 and 2015, with sample size ranged from 79 to 368. Table 1 describes the detailed characteristics of each study, including sample size, study origin, ethnicity, PF diagnosis measure, original diseases, and mean age of the 7 studies. Geographically, 3 were conducted in China; 1 in Korea, Britain, Spain, and Saudi Arabia. All pulmonary fibrosis patients were diagnosed by either HRCT and/or biopsy. Regarding original diseases, 2 studies differed from the others: Wei et al^[19]^ and EI-Gamel et al^[20]^ included pulmonary fibrosis patients with non-IPF disease, whereas the rest of studies involved patients all diagnosed as having IPF. To make the diagnosis of idiopathic pulmonary fibrosis, each study used the following diagnostic criteria: the American Thoracic Society, European Respiratory Society, the Japanese Respiratory Society, and the Latin American Thoracic Association consensus statement. In 5 studies, the mean ages of cases were found to be around 60 years and half of the included studies had age-matched controls, while Alhamad et al^[21]^ and Xaubet et al^[22]^ used younger people as their controls. Wei et al^[19]^ and EI-Gamel et al^[20]^ did not provide age and sex information of patients.

**Table 1:**
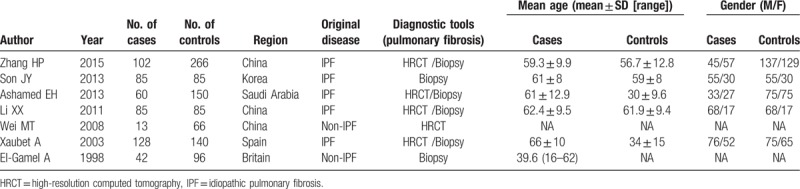
Main characteristics of studies included.

Average male-to-female ratios were found to be 1:1.56 for cases and 1.3:1 for controls, respectively. Two studies differed from the others regarding sex ratio: Li et al^[18]^ and Son et al^[17]^ included much less female patients than the other studies. Regarding original diseases, 2 studies were not the same as the others: Wei et al^[19]^ and EI-Gamel et al^[20]^ included SARS and pre-transplant pulmonary fibrosis, respectively, while the remaining studies enrolled IPF patients.

### 3.4 Results of meta-analysis

**Figure 2:**
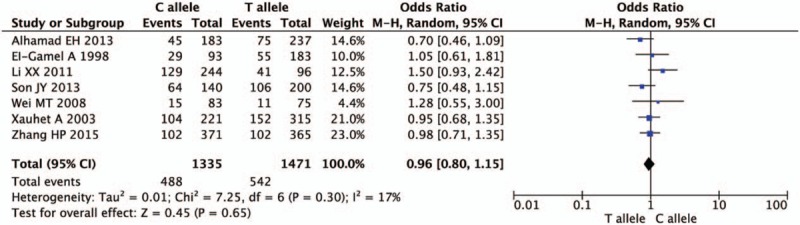
Forest plot of odds ratio from the random-effect model for allele comparison (T vs C). Each study is represented by a square whose area is proportional to the weight of the study. The overall effect from meta-analysis is represented by a diamond whose width represents the 95% confidence interval (CI) for the estimated effect size (OR). OR = odds ratio.

**Figure 3:**
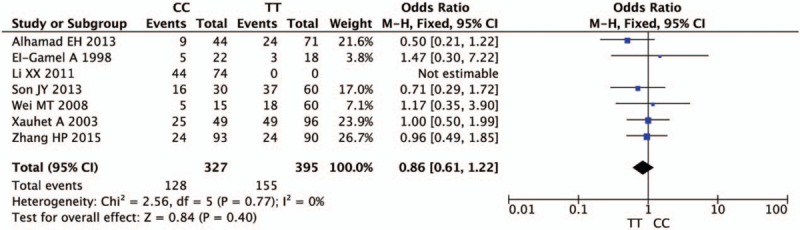
Forest plot of odds ratio from the fixed-effect model for homozygote comparison (TT vs CC). Each study is represented by a square whose area is proportional to the weight of the study. The overall effect from meta-analysis is represented by a diamond whose width represents the 95% confidence interval (CI) for the estimated effect size (OR). OR = odds ratio.

**Figure 4:**
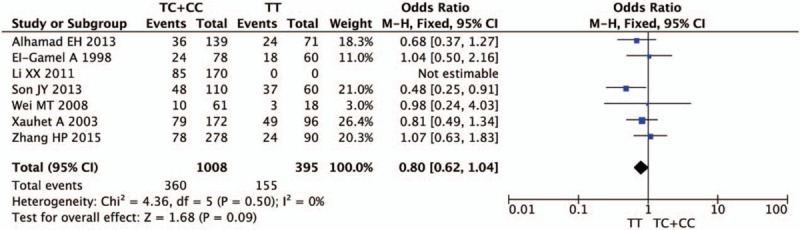
Forest plot of odds ratio from the fixed-effect model for dominant model (TT vs TC + CC). Each study is represented by a square whose area is proportional to the weight of the study. The overall effect from meta-analysis is represented by a diamond whose width represents the 95% confidence interval (CI) for the estimated effect size (OR). OR = odds ratio.

**Figure 5:**
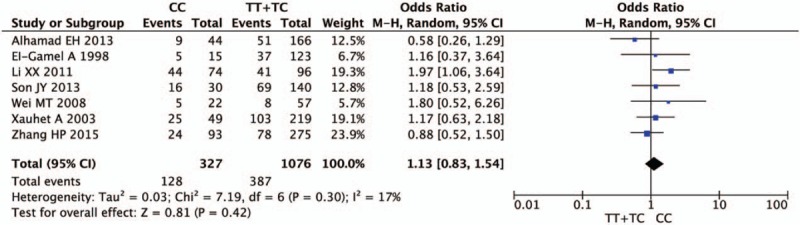
Forest plot of odds ratio from the fixed-effect model for recessive model (TC + TT vs CC). Each study is represented by a square whose area is proportional to the weight of the study. The overall effect from meta-analysis is represented by a diamond whose width represents the 95% confidence interval (CI) for the estimated effect size (OR). OR = odds ratio.

Figures 2–5 and Table 2 summarized the results of the meta-analysis. Overall, no significant association was found between TGF-β1 rs1800470 polymorphism and pulmonary fibrosis in any of the comparison models we used. The odds ratio (OR) and 95% confidence interval (CI) for each model were as follows: 0.96 [0.80, 1.15] in the allele comparison model (T vs C); 0.86 [0.61, 1.22] in the homozygote comparison model (TT vs CC); 0.80 [0.62, 1.04] in the dominant genetic model (TT vs TC + CC); 1.13 [0.83, 1.54] in the recessive model (TT + TC vs CC). To rule out the effect of ethnicity and original diseases, we subsequently performed subgroup analysis. There was no statistically significant association between TGF-β1 rs1800470 polymorphism and PF in any of the ethnic and disease subgroups (Table 2), except that there was a trend that the frequency of pulmonary fibrosis was less in genotype TT than that of other genotypes with respect to homozygote comparison model (TT vs CC: OR [95% CI] = 0.79 [0.52, 1.21]) and dominant model (TT vs TC + CC: OR [95% CI] = 0.75 [0.51, 1.10]) among Asians. Similarly, the pattern can be seen in the idiopathic pulmonary fibrosis patients, OR and 95% CI = 0.82 [0.56, 1.19] and 0.76 [0.55, 1.04] for homozygote and dominant model, respectively.

**Table 2:**
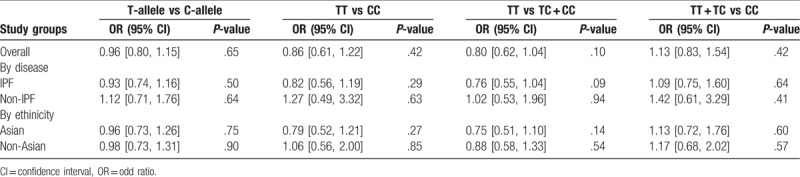
Main results in the total and subgroup analysis.

### 3.5 Publication bias

**Figure 6:**
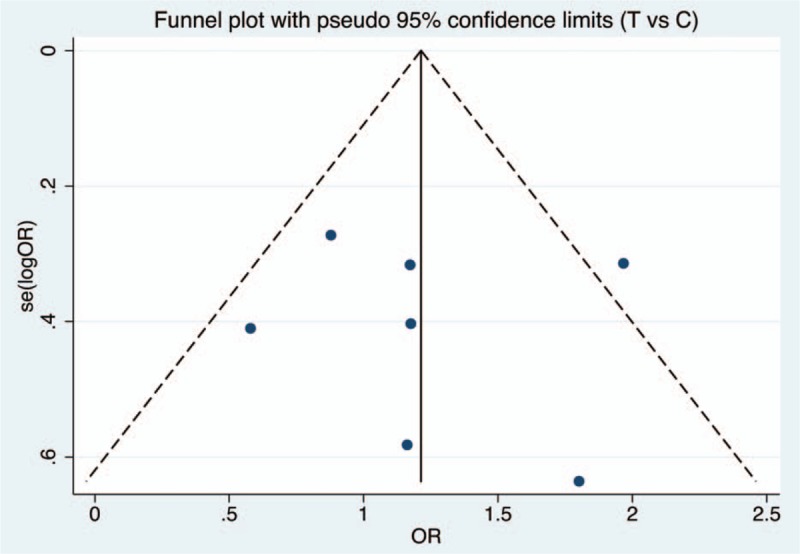
Funnel plot of publication bias with pseudo 95% confidence limits for allele comparison model.

**Figure 7:**
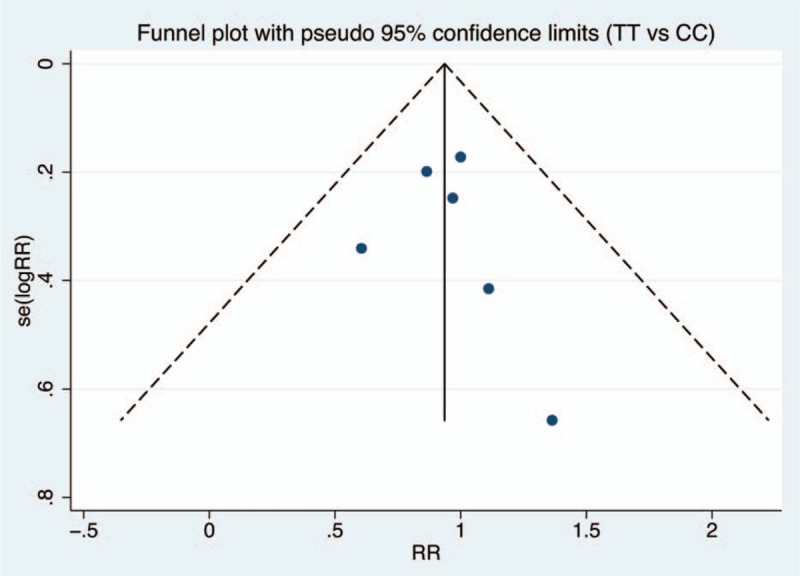
Funnel plot of publication bias with pseudo 95% confidence limits for homozygote model.

**Figure 8:**
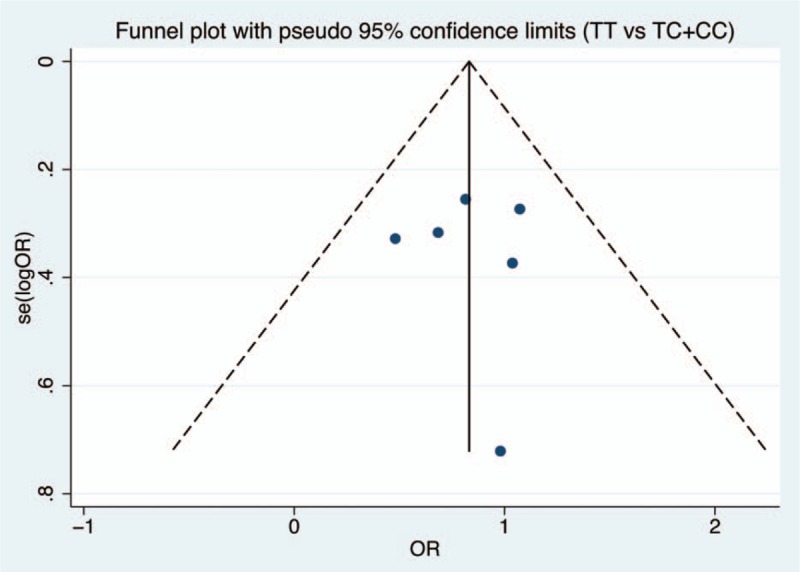
Funnel plot of publication bias with pseudo 95% confidence limits for dominant model.

**Figure 9:**
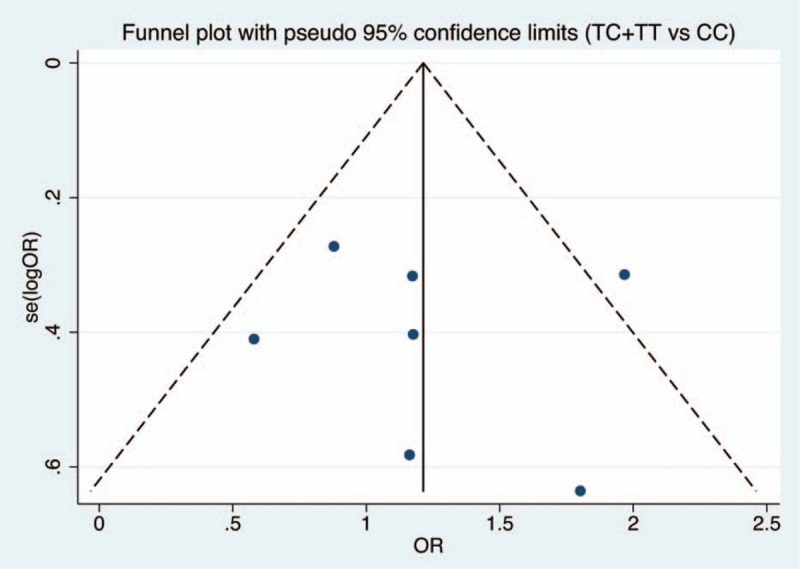
Funnel plot of publication bias with pseudo 95% confidence limits for recessive model.

Figures 6–9 and Table 3 show the publication bias for the 4 comparison models. All models did not present obvious publication bias, calculated by the Begg and Egger test (*P* > .05).

**Table 3:**
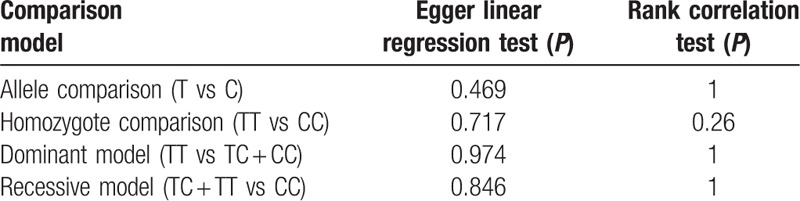
Assessment of publication bias.

## 4 Discussion

Association of TGF-β1 polymorphism with susceptibility to various diseases such as autoimmune disease, infectious disease, and cancer has been previously reported.^[14,23]^ Given the fact that the levels of TGF-β1 in blood or in injured lung were higher in patients with pulmonary fibrosis than that in healthy controls,^[24]^ multiple studies have reported conflicting results regarding the association of TGF-β1 polymorphism with pulmonary fibrosis. To our best knowledge, this is the first comprehensive meta-analysis to evaluate the association between TGF-β1 gene polymorphism and pulmonary fibrosis development. Although some studies reported association between them, our meta-analysis did not find any TGF-β1 rs1800470 polymorphism associated with susceptibility to pulmonary fibrosis. The present finding is consistent with the general trend from various pulmonary fibrosis genetic studies, which can be attributed to the polygenetic nature of the disorder.^[25,26]^ Further subgroup analysis and publication bias test (*P* > .05) provided additional support for the robustness of our results.

EI-Gamel et al^[20]^ were the first to report the role of TGF-β1 polymorphism in pre-transplant and allograft lung fibrosis. They found that the distribution of TGF-β1 rs1800470 alleles was similar in a normal healthy control group and in lung transplant recipients with pulmonary fibrosis. Son et al^[17]^ found that the TT genotype had a positive association with idiopathic pulmonary fibrosis development (*P* = .037), while Li et al^[18]^ demonstrated that the proportion of subjects with an CC genotype was significant higher in idiopathic pulmonary fibrosis group than that in control group subjects. However, several other studies could not find any noticeable association of TGF-β1 rs1800470 polymorphism with pulmonary fibrosis.^[19,21,22,27]^

Our meta-analysis included 515 patients and 888 healthy controls from 7 independent studies confirmed the absence of the association between TGF-β1 rs1800470 polymorphism and risk of pulmonary fibrosis development. This inconsistency might be attributed to the small sizes of the studies included in this meta-analysis. The maximum number of patients with pulmonary fibrosis was 128.^[22]^ In case of the report by Wei et al^[19]^ published in the year 2008, data of only 13 patients with SARS recovery-related lung fibrosis were used in their analysis. The lack of association might also be attributed to the polygenic contribution of the disorder, which means that individual gene only contributes a modest effect, but collectively, they have a cumulative effect on the likelihood of developing the illness.^[25,28]^

We performed subgroup analyses on ethnicity and original disease to exclude potential sources of heterogeneity among the included studies. However, we could not find any statistically significant association in the subgroups analysis. A slightly less frequency of pulmonary fibrosis was observed among IPF patients with TT genotype (*P* = .09), which might be attributed to a single study that had reported a relatively strong association, rather than a low frequency of this genotype in IPF patients in general.

As with all meta-analysis, several limitations merit consideration in our study. First, there is a lack of broader coverage on the investigation of other TGF-β1 polymorphisms related to pulmonary fibrosis risk, thus hindering our ability to conduct a wider spectrum of analysis encompassing more SNPs. The present meta-analysis was only able to collect data on one most studied SNP to perform the effect size calculation. There are other relevant TGF-β1 SNPs that have been implicated in pulmonary fibrosis susceptibility; one example is the SNP rs1800471, which has been associated with fibrosis in lung transplant recipients,^[20]^ but the locus was omitted in our analysis owing to a lack of sufficient studies (i.e., <3 studies that provided reusable genetic information). Second, in case-control design studies, population stratification analysis is often necessary to warrant that samples in cases and controls share common genetic characteristics other than the SNPs of interest.^[29]^ Thus, some of our results may be hampered by this shortcoming. For example, some studies didn’t provide smoke status information of subjects. Differences in smoke ratio are of particular importance, since smoke people are more likely to predispose to pulmonary fibrosis.^[30,31]^ Owing to the lack of genotype information by smoke status, we were unable to do a subgroup analysis. Third, in view of the relatively small sample size and the limited study number, especially in the subgroup of the non-IPF disease where only 2 studies were included, the power used to detect the real difference between cases and controls may not be very strong. Finally, PF is a multifactorial disease, the potential interaction of genetic–genetic or genetic–environmental factors may influence the process of PF.

In conclusion, our study found that the existing literature does not suggest the association of TGF-β1 rs1800470 polymorphism with pulmonary fibrosis. Considering the polygenic effect on pulmonary fibrosis, a large number of case-control studies with the information on other related genetic polymorphisms could provide reliable evidence for the role of TGF-β1 polymorphism with respect to susceptibility to pulmonary fibrosis.

## Author contributions

**Conceptualization:** Lili Xin, Miao Jiang.

**Data curation:** Lili Xin, Miao Jiang, Guangbao Su.

**Methodology:** Lili Xin, Miao Xie, Hui Chen, Xiao Liu.

**Supervision:** Jiening Gong

**Validation:** Lili Xin, Miao Jiang, Guangbao Su, Miao Xie, Hui Chen, Xiao Liu, Jiening Gong.

**Writing – original draft:** Lili Xin.

**Writing – review & editing:** Muge Xu, Geng Zhang.

**Conceptualization:** Lili Xin.

**Data curation:** Lili Xin, Miao Jiang, Guangbao Su.

**Methodology:** Lili Xin, Miao Xie, Hui Chen.

**Supervision:** Jiening Gong.

**Validation:** Lili Xin, Xiao Liu.

**Writing – original draft:** Lili Xin.

**Writing – review & editing:** Jiening Gong, Muge Xu, Geng Zhang.
